# Dietary *Boswellia serrata* Acid Alters the Gut Microbiome and Blood Metabolites in Experimental Models

**DOI:** 10.3390/nu14040814

**Published:** 2022-02-15

**Authors:** Cassandra Suther, Lauren Daddi, Suresh Bokoliya, Hunter Panier, Zhongmao Liu, Qingqi Lin, Yanhui Han, Kun Chen, Matthew D. Moore, Yanjiao Zhou

**Affiliations:** 1 Department of Food Science, University of Massachusetts, Amherst, MA 01003, USA; csuther@umass.edu (C.S.); yanhuihan1990@gmail.com (Y.H.); 2 Department of Medicine, University of Connecticut Health Center, Farmington, CT 06030, USA; lauren.daddi@uconn.edu (L.D.); bokoliya@uchc.edu (S.B.); hpanier@uchc.edu (H.P.); 3 Department of Statistics, University of Connecticut, Storrs, CT 06269, USA; zhongmao.liu@uconn.edu (Z.L.); kun.chen@uconn.edu (K.C.); 4 Department of Computer Science and Engineering, University of Connecticut, Storrs, CT 06269, USA; qingqi.lin@uconn.edu

**Keywords:** gut microbiome, *Boswellia serrata*, metabolomics, *Akkermansia*

## Abstract

*Boswellia serrata*, commonly known as frankincense, has been used for centuries as a natural anti-inflammatory and anti-microbial remedy for many illnesses. However, the effect of the bioactive ingredient of it, 3-O-acetyl-11-keto-b-boswellic acid (AKBA), on both the gut microbiome and blood metabolites, is not known. In this study, we observe the effect of this isolated active ingredient orally on both male and female mice. Gut microbiota and blood metabolites were determined at the beginning and end of a 14-day consumption period. AKBA significantly decreased gut bacterial richness in male mice, and had no effect on female mice. *Akkermansia muciniphila*, associated with weight loss and anti-inflammation, was found to be significantly increased in both male and female mice, along with an increase in *Bifidobacterium* in female mice. *Akkermansia muciniphila* and *Bifidobacterium* were plated on media containing varying levels of AKBA (0%, 0.001%, 0.01%, and 0.1%). All concentrations of AKBA completely inhibited growth of *Akkermansia muciniphila* but had no effect on *Bifidobacterium*. Several blood metabolites differed with AKBA between both males and females. These results show the potential benefits of dietary *Boswellia serrata* on the modulation of gut microbiome composition, along with differences between sexes.

## 1. Introduction

The gut microbiome, a collection of microbiota and their genetic contents from the gastrointestinal tract, has been shown to play an important part in health [1,2]. Gut microbiome dysbiosis is thought to be linked to the development of disease and weight gain [1,3]. Diet has a profound effect on the configuration of the gut microbiome. As a modifiable target, modulation of the gut microbiome through dietary intervention, including prebiotics or probiotics, have seen an increase in both research interest and product development in past years [4].

Gibson and Roberfroid first defined prebiotics as “selectively fermented ingredients that allow for specific changes, both in the composition of and/or activity in the gastrointestinal microflora that confer benefits upon hosts well-being and health” [4]. Other criteria have been added to the definition of prebiotic, including being safe for ingestion and resistant to gastric acidity [4]. Dietary fiber has been the most recognized prebiotic and includes inulin, fructo-oligosaccharides, and other oligosaccharides [4]. However, other non-fiber substances are increasingly being recognized as having prebiotic effects. Polyphenols are exhaustively metabolized by gut bacteria and are thought to produce beneficial by-products [5]. Other studies have found rhubarb extract, a Chinese herb containing no fiber or polyphenols, to modulate the gut microbiome to increase *Akkermansia muciniphila* [6].

*Boswellia serrata* is a branching tree that grows in the dry regions of India and the Middle East. These trees contain a gum resin commonly known as frankincense or olibanum [7]. The use of *Boswellia serrata* varies, being utilized in cosmetics, materials, foods, and pharmaceutical products. For centuries, these gum resins have been used as a remedy for a variety of inflammatory and bacterial diseases. The active compounds are thought to be the boswellic acids (BAs) (pentacyclic triterpenic acids) [7]. BAs are commonly isolated and used in a variety of disease models and human research, including asthma, arthritis, and several cancers [8,9,10]. In recent years, BAs have been established as a multitargeting agent, modulating several targets, including enzymes 5-Lipoxygenase (5-LOX), cyclooxygenases 1 (COX-1), growth factors (Vascular endothelial growth factor), IkappaB kinase (I-κB kinases), transcription factor Signal Transducer and Activator of Transcription 3 (STAT3), Death receptor 4 (DR4), and others related to the survival and proliferation of cells (Myeloid leukemia 1) [10]. Notably, BAs can inhibit 5-LOX and COX-1 activities by directly binding activity sites of these enzymes [11,12]. The most potent and researched BA is 3-O-acetyl-11-keto-b-boswellic acid (AKBA). However, the effects of AKBA ingestion on gut health has not been explored.

It has been well-established that AKBA has anti-bacterial and antifungal activity [13]. However, the exact mechanism of antimicrobial action is not clearly established, as multiple studies have contradicting conclusions based on Gram stain, as inactivation does not seem to correlate with Gram status [14,15,16].

In this study, we investigated the effect AKBA on the gut compositions of both healthy male and female mice. We also examined the antibacterial effect of AKBA through in vitro assays. Along with this, as changes in the gut microbiome are linked to changes in circulating metabolites within the body [17], we also investigated differences in blood metabolites. Isolated AKBA itself contains no fiber or polyphenols and any changes to the gut microbiome would be due to a newly reported compound. To our knowledge, this is the first paper to report the effects of AKBA on the gut microbiome and the changes this causes in blood metabolites.

## 2. Materials and Methods

### 2.1. Animals

Eight-week-old BALB/cJ male and female mice were purchased from the Jackson Laboratory (Bar Harbor, ME, USA) and housed in plastic cages with corncob bedding at the University of Connecticut Health Center (Farmington, CT, USA). Animal rooms maintained a 12-h light/dark cycle and were pathogen free. Mice were fed a 18% protein rodent diet (18.6% protein, 6.2% fat, 44.2% carbohydrate, 3.5% soluble fiber, 14.7% total fiber, and 5.3% ash) purchased from Envigo, (Indianapolis, IN, USA) and supplied autoclaved water. Bedding was mixed between all cages a week prior to start of experiment to homogenize the gut microbiomes within the same sex. Afterwards, mice within the same gender were randomized into AKBA groups (10 mice per group/5 mice a cage) and controls (5 mice per cage/5 mice per cage). For the AKBA group, mice were gavaged with 200 μL of 100 mg/kg AKBA (pH 7) (AquaLOX, PLT Health Solutions, Morristown, NJ, USA) once daily for 14 days (AKBA male *n* = 10, AKBA female *n* = 10). One cage from AKBA group was designated as AKBA1 and the other cage as AKBA2. For control groups, mice were gavaged with 200 μL PBS daily for 14 days (control males *n* = 5, control female *n* = 5). Because no previous studies have investigated effects of AKBA on the gut microbiome, 5–10 mice/group were used; however, effect size and sample size could not be determined precisely.

Animal protocol 102063-0522 was approved by the University of Connecticut Health Center IACUC board.

### 2.2. Sample Collection, DNA Extraction, and 16S rRNA Gene Amplification and Sequencing

Fresh fecal pellets were collected upon defecation on day 0 and day 14, and stored in sterile RNA-/DNA-/RNAse-/DNAsefree microcentrifuge tubes at the beginning and end of experiment. Collected pellets were placed at −80 °C for storage following collection. DNA from pellets were extracted using PowerSoil DNA Isolation Kit (Qiagen, Hilden, Germany, Eur) according to the manufacturer’s protocol. Amplicon library preparation and sequencing were performed by the Microbial Analysis, Resources, and Services Facility at University of Connecticut, Storrs. Briefly, DNA extracts were quantified using the Quant-iT PicoGreen kit (Invitrogen, Waltham, MA, USA). In brief, bacterial 16S rRNA gene was amplified using 30 ng extracted DNA as template. The V4 region was amplified using 515F and 806R primers with Illumina adapters and dual indices (eight base pairs) [13]. Samples were amplified in triplicate 15 uL reactions using Go-Taq DNA polymerase (Promega) with the addition of 3.3 µg BSA (New England BioLabs). To overcome inhibition from host DNA, 0.1 pmol primer without the indexes or adapters was added to the mastermix. The PCR reaction was incubated at 95 °C for 3.5 min, then 30 cycles of 30 s at 95.0 °C, 30 s at 50.0 °C, and 90 s at 72.0 °C, followed by final extension of 72.0 °C for 10 min. PCR products were pooled for quantification and visualization using the QIAxcel DNA Fast Analysis (Qiagen). PCR products were normalized based on the concentration of DNA from 250–400 bp then pooled using the epMotion 3075 liquid handling robot. The pooled PCR products were cleaned using Omega Bio-Tek Mag-Bind Beads according to the manufacturer’s protocol using 0.8x beads to PCR product. The cleaned pool was sequenced on the MiSeq using the v2 2x250 base pair kit (Illumina, San Diego, CA, USA).

### 2.3. Processing of the 16S rRNA Gene Sequences

Raw 16S rRNA gene sequences were initially processed by BaseSpace software (Illumina, San Diego, CA, USA). One mismatch in primer and zero mismatch in barcodes were applied to sample deconvolution. Deconvoluted sequences were further processed using the DADA2 data processing pipeline with default parameters to obtain amplicon sequence variants (ASVs) [18]. Final taxonomic assignment was conducted using RDP-classifier (v2·11) (Michigan State University, East Lansing, MI, USA) with a confidence value of 0.5 as cut-off. Reads with <0.5 confidence of classification was considered to be “unclassified” at a given taxonomical level.

### 2.4. Statistical Analysis of 16S rRNA Gene Data

ASV counts were converted to relative abundance and agglomerated at the phylum and genus levels using the Phyloseq R package to visualize the microbiome composition in a stacked bar plot. All 10 phyla were included in the barplot at phylum level, while the most abundant 24 genera were displayed in the barplot at genus level, including all additional genera grouped together in a 25th category as “other”. Sample reads were rarefied to 10,000 reads to account for uneven sampling depth.

Alpha diversity was determined with the Richness and Shannon diversity metrics. Wilcoxon sum rank test was performed to compare alpha diversity of stool microbiome between male and female mice from AKBA-treated and control groups on day 0 and day 14. We also performed linear mixed regression with alpha diversity as the response variable, treatment, time points, and their interactions as fixed effects, and individual mice as a random effect.

Beta diversity was visualized by Principal Coordinates Analysis (PCoA) using the Bray–Curtis dissimilarity metrics. Microbiome community structure difference between two groups were determined by conducting PERMANOVA on Bray–Curtis dissimilarity and Jaccard indices using the Adonis function in the Vegan R package. Differential analysis of the relative abundance of various taxonomy in the microbial communities at the phylum and genus level was performed using LEfSe based on Linear discriminant analysis Effect Size (LDA) [19]. The effect size of differentially represented genera were represented by LDA scores. An adjusted *p* value < 0.05 by false discovery rate (FDR) was considered statistical significance.

To identify relationships between baseline microbiome (*Akkermansia* and *Bifidobacterium*) on day 0 and the microbiome on day 14 in the AKBA-treated group, we performed a linear regression with cage as a covariate.

All plots were created with “ggplot2”, and all analyses were conducted in RStudio version 4.1.0 (RStudio, Boston, MA, USA)

### 2.5. Blood Metabolome Analysis with Mass Spectrometry

Mice were bled via cheek bleeding at the beginning and end of the experiment. Blood was collected, allowed to clot at room temperature for an hour, and spun down at 500 g. The resulting serum was collected and stored at −80 °C until further analysis. For mass spectrometry preparation, serum samples were vortexed with 80% chilled aqueous methanol in a ratio of 1 (serum):60 (methanol) (*v*/*v*). Protein was precipitated and centrifugated (14,000× *g*, 4 °C, 10 min). The supernatant was collected and dried under a vacuum. The dried residues were reconstituted with 50% methanol and centrifuged (14,000× *g*, 4 °C, 15 min). Ultraperformance liquid chromatography-mass spectrometry (UPLC-MS) was performed at the University of Massachusetts Amherst (Amherst, MA, USA) mass spectrometry facility to analyze the mouse serum for untargeted metabolomics. An equal volume of each serum sample was pooled to prepare the quality control (QC) sample. Five identical QC sample runs were conducted prior to running test samples, and one QC sample run was performed for every eight sample runs throughout the experiment. Acquity UPLC HSS T3 column (2.1 mm × 100 mm, 1.8 µm, Waters Co., Milford, MA, USA) was used to obtain chromatographic separation by injecting 5 µL aliquots of each sample. The column was maintained at 40 °C, with a flow rate of 0.5 mL/min. Solvent A contained 95% water with 5% acetonitrile and 0.1% formic acid and solvent B contained 100% ACN with 0.1% formic acid. The gradient started at 2% of solvent B and linearly increased to 95% at 8 min; once at 95%, solvent B was held for 2 min. Between the runs, the column was equilibrated at 2% of solvent B for 5 min. MS was conducted using the Thermo Fisher Orbitrap-Fusion in negative electrospray ionization mode at the detection range of 120–1000 *m*/*z* with 60,000 full width at half maximum resolution. The following conditions were used for MS: spray voltage, 3500 V; sheath gas flow rate, 15 (arbitrary units); auxiliary gas flow rate, 6 (arbitrary units); sweep gas flow rate, 3 (arbitrary units); vaporizer temperature, 275 °C; and ion transfer tube temperature, 325 °C.

### 2.6. Identification of Differently Abundant Metabolites and Pathway Activity

The raw data were analyzed by MetaboAnalyst (https://www.metaboanalyst.ca/ (accessed on 3 February 2022)). The metabolites of interest were identified by matching their profiles relating to accurate mass values, retention time, isotope peak matching, mass-to-charge ratio, and fragment information against different databases, including ChemSpider (http://www.chemspider.com (accessed on 3 February 2022)) and KEGG global metabolic network (https://www.genome.jp/kegg/ (accessed on 3 February 2022)). Pathway activity data were filtered by interquartile range, normalized by the median, log transformed, and set to an FDR cut off *p*-value of <0.05 using Fisher’s exact test with pathways containing < 3 metabolites using Mummichog v2. Principal Coordinates Analysis (PCoA) were created with “ggplot2” in Rstudio version 4.1.0. PERMANOVA was preformed between groups to a set *p*-value of <0.05.

### 2.7. Bacterial Plating and Load Analysis

*Akkermansia muciniphila* (ATCC BAA-835) and *Bifidobacterium pseudolongum* (isolated and identified in stools from mice treated with AKBA for 14 days) glycerol stocks were thawed and streaked onto a modified brain heart infusion (BHI) media that contains yeast extract, hemin, vitamin K1, l-cysteine, and resazurin indicator. Following incubation for 24 h at 37 °C in anaerobic conditions (7% H_2_, 10% CO_2_, N_2_ balance), one colony was inoculated into 1 mL sterile BHI broth. Broth was incubated for 48 h at 37 °C in anaerobic conditions. Subsequent 1:10,000, 1:100,000, and 1:1,000,000 dilutions were made and plated on to BHI plates containing either 0% *w*/*v* AKBA, 0.001% *w*/*v* AKBA, 0.01% *w*/*v* AKBA, or 0.1% *w*/*v* AKBA. Agar plates were incubated for 48 h at 37 °C in anaerobic conditions. Individual colonies were counted.

A baseline mouse stool pellet was vortexed in 1 mL of sterile PBS. From that, 200 μL of stool slurry was added to six tubes of 5 mL modified BHI media (as mentioned above). Half the tubes received AKBA, for a final concertation of 0.1% *w*/*v*. Broth was incubated for 48 h at 37 °C in anaerobic conditions. Following incubation, 250 μL of broth was extracted using PowerSoil DNA Isolation Kit (Qiagen, Hilden, Germany) according to the manufacturer’s protocol. qPCR was then preformed using NEB Luna^®^ Universal qPCR Kit (New England Biolabs, Ipswich, MA, USA). A 16S rRNA gene primer targeting the V4 region was used [13]. Amplification was then performed for 2 min of 95 °C for enzyme activation, then 30 cycles at 95 °C for 30 s and 60 °C for 15 s. At least two reactions were performed per sample, with at least two separate reactions run. Averages were taken between groups.

## 3. Results

### 3.1. Baseline Microbiome Composition

**Figure 1:**
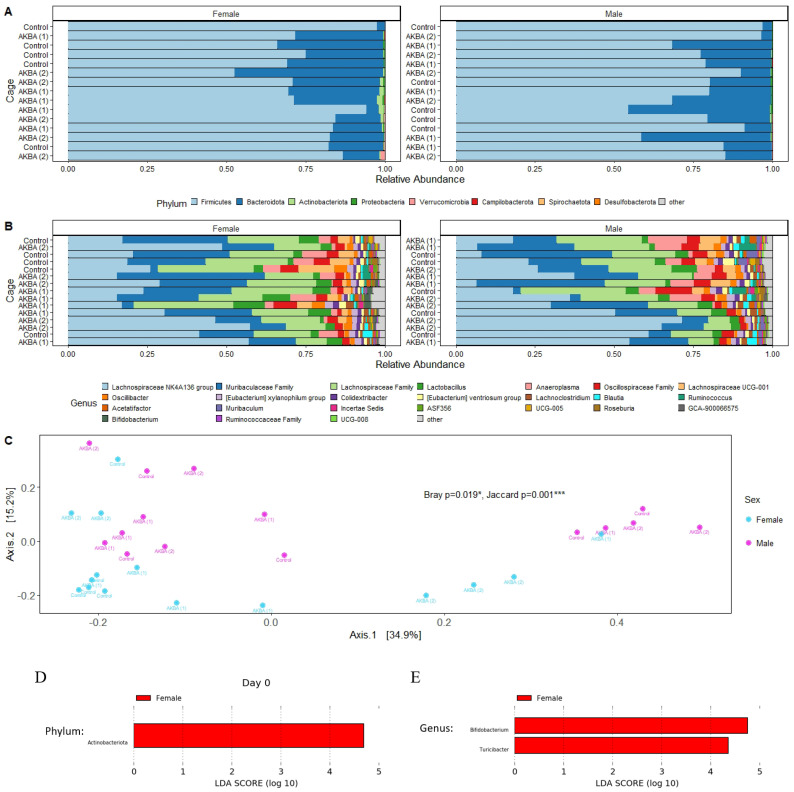
(**A**–**E**) Stool microbiome composition on the day 0 baseline. (**A**) Bar plots at phylum level for AKBA1 (cage 1 in Acetyl-11-keto-beta-boswellic acid (AKBA)-treated mice), AKBA2 (cage 2 in AKBA-treated mice), and control groups. There is a higher relative abundance of Actinobacteria in female mice at baseline. (**B**) Bar plots of the top 24 genera for AKBA1, AKBA2, and control groups. (**C**) Principal Coordinates Analysis (PCoA) plot using Bray–Curtis dissimilarity displaying strong clustering of female and male baseline microbiome samples. PERMANOVA results indicate significant differences using Bray–Curtis and Jaccard metrics. (**D**,**E**). Significant phylum and genera between male and female identified by linear discriminant analysis. Compared with control group and AKBA group, * *p* < 0.05, *** *p* < 0.001.

A total of 2,202,430 high-quality reads were obtained for an average of 36,707 reads per sample. Reads were clustered into 665 unique ASV. At the phylum, we found a higher relative abundance of Actinobacteria phyla in female mice compared to male mice at baseline (Figure 1A), with a linear discriminant analysis score of 4.80 (*p* < 0.05). (Figure 1D). At the genus level, we showed the top 24 most abundant genera in the AKBA-treated and control mice (Figure 1B). Linear discriminant analysis with LEfSe identified *Bifidobacterium* and *Turicibacter* were significantly higher in female mice compared to male mice (Figure 1E). The microbiome difference by gender was further supported by PERMANOVA statistical tests, which indicated significant differences between male and female mice at baseline using the Bray-Curtis (*p* = 0.019) and Jaccard (*p* = 0.001) distance metrics, as illustrated by a PCoA plot (Figure 1C).

Additional PERMANOVA testing was performed to determine any microbiome differences between the control and AKBA-treated groups, for male and female mice separately at baseline. No significant differences were found between AKBA groups and control group with either the Bray–Curtis or Jaccard metrics for male (*p* = 0.412, 0.423) and female (*p* = 0.138, 0.267) mice at baseline.

Based on these findings, it was chosen to analyze the male and female mice separately to compare differences in the microbiomes following AKBA or control treatment.

### 3.2. Modulation of Male Microbial Communities with AKBA Supplementation

**Figure 2:**
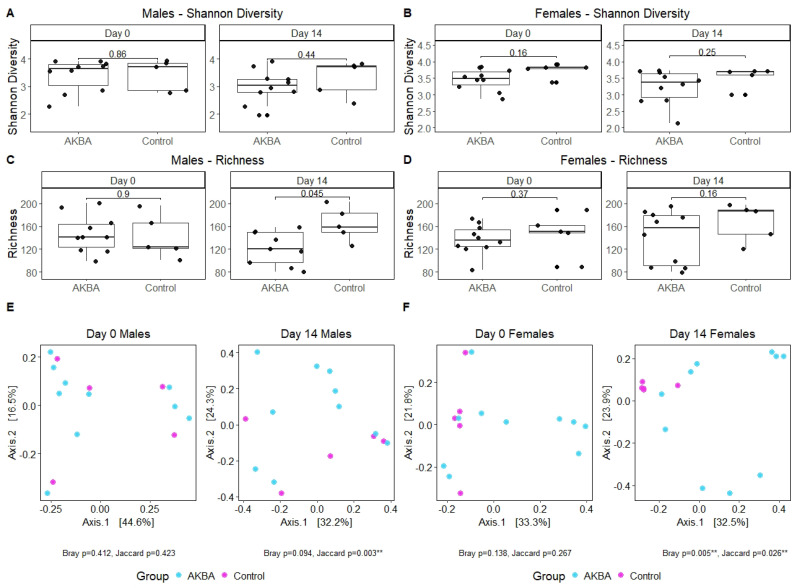
(**A**–**D**) Alpha diversity was determined for male and female mice using the Shannon and Richness metrics. Significant differences were found between male AKBA and control treatment groups in Richness on day 14. (**E**,**F**) PCoA plots using Bray–Curtis dissimilarity and PERMANOVA test to show strong clustering and indicate significant difference between treatment groups for male and female mice on day 14. Compared with control group and AKBA group, ** *p* < 0.01.

We first compared the bacterial diversity difference between the AKBA-treated group and control group at either baseline or on day 14. Shannon diversity was not significantly at both time points (Figure 2A). Bacterial richness was significantly lower in male mice after 14 days of AKBA treatment, compared with control male mice on day 14 (Wilcoxon, *p* = 0.045) (Figure 2C). Linear mixed model with individual mice as random effect and treatment, time points, and their interaction as fixed effect showed that richness changed differently over time in the AKBA group and control group in male mice (*p* = 0.02), with richness significantly decreased from baseline to day 14 in the AKBA group (*p* = 0.005). Principal Coordinates Analysis (PCoA) using Bray–Curtis dissimilarity displayed strong clustering by treatment group on day 14. PERMANOVA testing indicated significant differences between treatment groups on day 14 with Jaccard (*p* = 0.003) distance metrics, and marginal significant difference with Bray–Curtis (*p* = 0.094) dissimilarity (Figure 2E).

**Figure 3:**
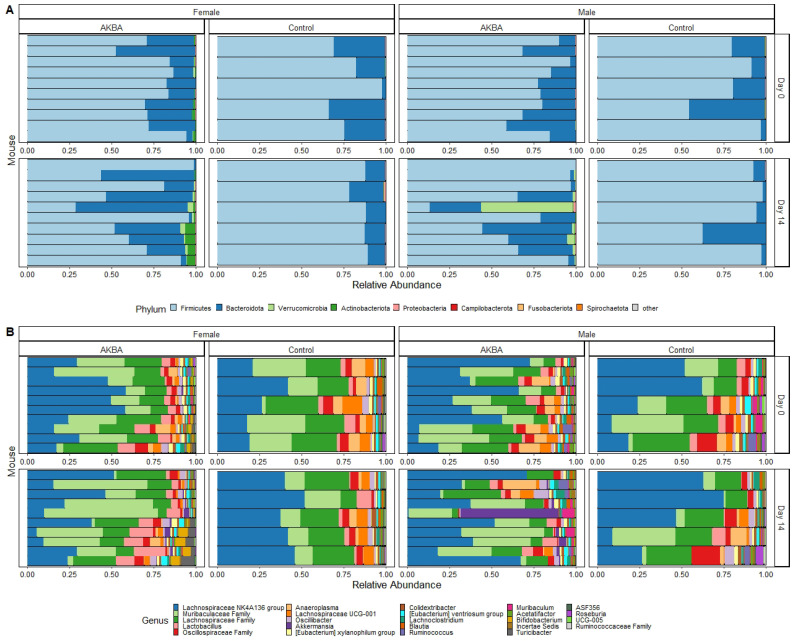
(**A**,**B**) Bar plots showing relative abundance of phyla (**A**) and genera (**B**) in the AKBA-treated and control male and female mice.

**Figure 4:**
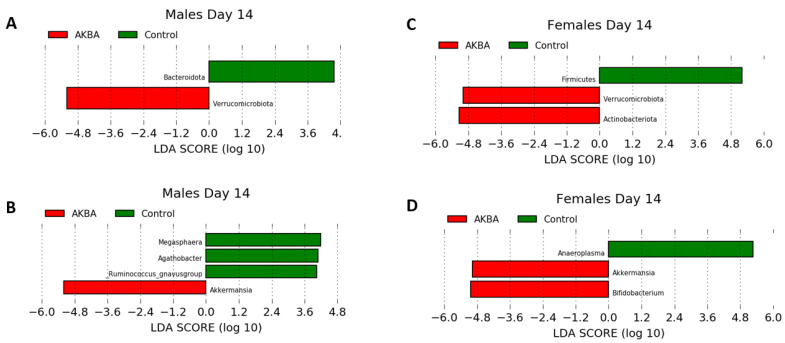
(**A**–**D**) LDA effect size showing differential microbiota at the phylum level for male (**A**) and female (**C**) mice, and at the genus level (**B**,**D**) following 14 days treatment in AKBA and control groups.

Further analysis to evaluate AKBA-specific taxonomy differences in the treatment group microbiomes was performed. Bar plots of relative abundance of top taxa at the phylum and genus level indicated an increase in *Akkermansia* (phylum Verrucomicrobia) in AKBA-treated mice (Figure 3A,B). LEfSe testing for differential abundance at the phylum and genus level further verified this finding (Figure 4A,B). Notably, there was a high level of variation in the increase of *Akkermansia* among one male mice, which had no *Akkermansia* at baseline but had a 53% abundance increase following treatment. In addition, a decrease in abundance of the genera *Megasphaera*, *Agathobacter*, and *Ruminococcus gnavus* group was observed with AKBA treatment, as these genera were significantly higher in abundance in the control male mice (LDA scores of 4.18, 4.08, and 4.04, respectively). Unlike female mice, male mice contain no detectable *Bifidobacterium* at baseline, with only four mice showing limited relative abundance after treatment (0.01–0.4%)

### 3.3. Modulation of Female Microbial Communities with AKBA Supplementation

There were no significant differences in alpha diversity between AKBA group and control group at the baseline time point, or post-AKBA supplementation (Figure 2B,D). Alpha diversity in both groups were not changed significantly from baseline to day 14 in either group. PCoA using Bray–Curtis dissimilarity showed strong clustering by treatment group on day 14, and PERMANOVA tests yielded significant results with Bray–Curtis dissimilarity (*p* = 0.005) (Figure 2F) and Jaccard (*p* = 0.026) distance metrics.

Bar plots of relative abundance appear to show an increase in the Actinobacteria phylum in AKBA-treated mice on day 14 (Figure 3A). Further LEfSe analysis revealed a significantly higher presence of Verrucomicrobia and Actinobacteria phyla on day 14, and *Akkermansia* and *Bifidobacterium* genera (LDA scores of 4.96 and 5.04, respectively), along with a significantly lower presence of *Anaeroplasma* (LDA score of 5.26) (Figure 4D).

### 3.4. Baseline Bifidobacterium and Its Response to AKBA Treatment

*Akkermansia* and *Bifidobacterium* were found significantly enriched in female mice following 14 days of AKBA treatment; however, only *Akkermansia* was found significantly enriched in male mice (Figure 4). To determine whether the abundances of these bacteria at the baseline were associated their response to AKBA treatment on day 14, we conducted a linear regression analysis with the relative abundances of these bacteria at baseline and on day 14 after controlling for cages. We did not find any association between the abundances of *Bifidobacterium* and *Akkermansia* at baseline and day 14 in female mice in the AKBA group after controlling for cages. No association was found for male mice baseline and endpoint *Akkermansia* after AKBA treatment.

### 3.5. AKBA Displays an Antimicrobial Effect on Overall Bacteria Load and Akkermansia but No Effect on Bifidobacterium In Vitro

Due to the observation of increased relative abundances of *Akkermansia* and *Bifidobacterium* after AKBA treatment, we further tested whether AKBA has any direct effect on the growth of these bacteria in vitro. We plated *Akkermansia muciniphila* (Gram−) and *Bifidobacterium pseudolongum* (Gram+) in the presence of different concentrations of AKBA (*w*/*v)* in BHI plates (0%, 0.001%, 0.01%, and 0.1%). Interestingly, *Akkermansia* only grew in control plates containing 0% AKBA (108 ± 4 at 10^−5^ dilution), showing strong inhibition at even low levels of AKBA. By contrast, *Bifidobacterium* growth was not affected by any AKBA concentrations.

Next, we wanted to check AKBA’s overall effect on bacterial load. After inoculating mouse stool in either BHI or BHI + AKBA, we found a significant decrease in Cq values following qPCR. The BHI control group had an average Cq value of 16.16 ± 0.44, while the BHI + AKBA had a Cq value of 21.11 ± 1.01. Extraction, broth, and qPCR controls were negative.

### 3.6. AKBA Induces Changes in Circulating Blood Metabolites in Both Male and Female Mice

**Figure 5:**
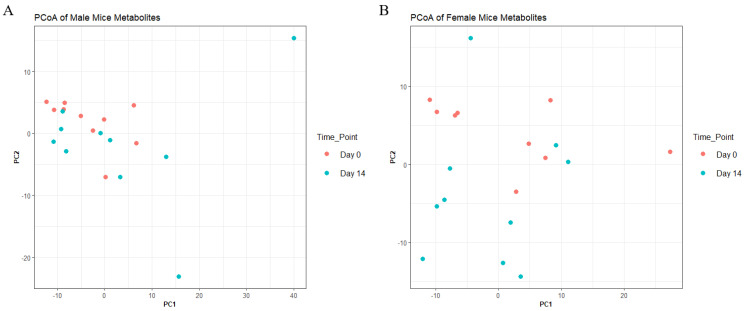
Principal coordinate analysis of blood metabolites in AKBA−treated mice on day 0 and day 14 for male (**A**) and female (**B**) mice.

To understand metabolite changes before and after 14-day treatment with AKBA, we performed untargeted MS and identified 397 metabolomic features for males and 406 metabolomic features for females. PCoA plots of metabolomic communities are shown (Figure 5). However, at the whole metabolome level, PERMANOVA analysis showed no significant difference between groups (*p*-value > 0.05, PERMANOVA). Feature level change was observed (adjusted *p*-value < 0.05, Wilcoxon) for both male and female mice. Males saw 4 significant feature changes (2 increases and 2 decreases following treatment) and females saw 12 changes (10 increases and 2 decreases following treatment) (Table S1). One metabolite change with an m/z and retention time of 163.0594_184.36 (Possible formula C_4_H_10_N_3_O_4_ or C_10_H_12_S) was found decreased between both groups. All metabolite feature change possible identities are shown in Table S1. Control mice from both sexes had no significant changes.

Prediction of pathways activities directly from LC-HRMS peaks was conducted using Mummichog (v2.0) in MetaboAnalyst 3.0. Activity in two pathways was predicted for female mice at a cut off *p*-value of <0.05; specifically, steroid biosynthesis (compounds hits 1/41, Fisher’s exact test, *p* = 0.08) and primary bile acid biosynthesis (compound hits 1/46, Fisher’s exact test, *p* = 0.08). Calcitetrol, which is known to stimulates intestinal calcium transport, and 3beta,7alpha-Dihydroxy-5-cholestenoate, a derivative from a bile acid, were shown to change with the AKBA supplementation. No pathways were identified in male or control mice.

## 4. Discussion

Acetyl-11-keto-beta-boswellic acid consumption altered the gut microbiome composition of both healthy male and female BALB/cJ mice. Interestingly, only *Akkermansia* was affected in male mice given AKBA, increasing throughout. However, *Bifidobacterium* were increased, along with *Akkermansia,* in female mice fed AKBA. The increase in *Bifidobacterium* in females, while not in males, could potentially be gender specific. Female mice are thought to naturally have higher counts of *Bifidobacterium* in the gut [20]. However, it should be noted the higher abundance of *Bifidobacterium* in female mice at baseline vs. male mice. It is also important to note the difference in relative abundance of *Bifidobacterium* between cages within female mice.

*Bifidobacterium* is a probiotic bacterium that is abundant in the guts of children during the first year of life, and has been detected in lower levels in adults. *Bifidobacterium* is known to secrete beneficial short chain fatty acids and lower inflammation [20] Turicibacter (part of the Firmicutes phylum) is commonly found in the intestines of animals [21]. *A. muciniphila* is a mucin-degrading, Gram-negative, oval-shaped, non-motile, and strictly anaerobic bacteria. It makes up around 0.5–5% of the total microbial composition of the human gut and its population has been correlated with a reduction in obesity, type 2 diabetes, and inflammatory bowel disease [22,23]. *A. muciniphila* is thought to be a “next-generation probiotic”, meaning it displays characteristics that are suspected to promote health, but is not part of the lactic acid group of probiotics that have been heavily studied [24]. As this study was conducted in healthy, young mice, future work should investigate the potential benefits of AKBA in disease and aging models. Many diseases, which AKBA has been traditionally used to treat (allergies and cancers), are known to have dysbiosis of the microbiome. *A. muciniphila* and *Bifidobacterium* have been shown to be negatively correlated with asthma and are thought to help protect against different forms of cancer [25,26,27,28].

*Akkermansia* and *Bifidobacterium* can be increased through changes in diet, with increased dietary fiber or possibly increases in polyphenols. However, the supplement of AKBA administered in this study contains neither of these, suggesting a different mechanism of action for the increase in these potentially beneficial bacteria than previously reported. To further test AKBA’s promotional effect on *Akkermansia* and *Bifidobacterium,* the bacteria were plated on different concentrations of AKBA. Interestingly, AKBA completely inhibited the growth of *A. muciniphila* at all concentrations tested, but had no effect on *Bifidobacterium.* AKBA has been used as an antimicrobial in different applications (skin, nails, and teeth) [14,15,16]. However, the exact mechanism of antimicrobial action of AKBA is not well characterized. Seeing as *A. muciniphila* is Gram negative (inhibited) and *Bifidobacterium* is Gram positive (not inhibited), one could suspect that Gram status may influence sensitivity to AKBA; however, previous studies have demonstrated the antimicrobial effect of AKBA on an array of Gram-positive and Gram-negative bacteria and fungi [14,15,16]. In this study, we found the bacterial load of a stool culture to be significantly lowered with the addition of AKBA. Larger concentrates of AKBA may be needed for different bacteria as well. It should be noted that the conditions found in vivo (GI tract) and in vitro (BHI plates) are vastly different environments and may affect bacterial growth conductions.

Overall richness was not affected by AKBA consumption in female mice, but was found to decrease significantly in males. A decrease in recorded ASV is not surprising, as AKBA displayed antimicrobial effects in this study and previous reports; however, more bacteria residing in females may be resistant to AKBA’s activity. Differential sex-dependent baseline microbiomes may affect these endpoint results and should be considered for gender-specific nutrition.

Although AKBA inhibited *A. muciniphila* in vitro, it is possible AKBA removed other bacteria in the gut, enabling *A. muciniphila* to opportunistically grow. When less bacteria are present surrounding the mucin layer of the colon, more mucin is available for *A. muciniphila* to thrive on, increasing the relative abundance [22]. It is also possible that the antimicrobial impact of AKBA has less effect on the bacteria in vivo near the mucin layer of the gut compared to bacteria in the lumen, resulting in a relatively higher abundance of mucin residents, such as *Bifidobacterium* and *Akkermansia.*

*Bifidobacterium* levels in the gut have been reported to be inversely correlated with inflammation in disease models [1]. The bacteria itself is thought to exhibit anti-inflammatory properties [20]. AKBA is well known for its own anti-inflammatory properties. From this, it begs the question if an increase of *Bifidobacterium* is a direct effect from the body’s responses to decrease in inflammation, or if the increase in *Bifidobacterium* is a mediator of AKBA’s anti-inflammatory properties. Further research should be conducted to further elucidate the role of AKBA and *Bifidobacterium* on the anti-inflammatory effects of AKBA consumption observed here.

The blood metabolomics in mice changed after 2 weeks of AKBA supplement, with only 4 metabolites changing in males and 12 metabolites changing in females. However, both did share one metabolite change with an m/z and retention time of 163.0594_184.36. Most of the feature-level compounds found from our untargeted MS analysis were unidentifiable, either due to no results in the database used or there being too many potential candidates to confidently identify a compound. Mummichog analysis identified two possible compound changes involved in steroid biosynthesis and primary bile acid biosynthesis in females. Changes in the gut microbial community can lead to changes in bile acids, with reduced bile acid levels in the gut being associated with bacterial overgrowth and inflammation [29].

Despite interesting and innovative findings in our study, there are several limitations. First, our findings were based on an unbalanced design, which led to less statistical power when compared to a balanced design. Second, the study is only conducted using one mice strain, one AKBA dosage, and one intervention course. It would be interesting to test the effect of AKBA on the microbiome using different strains of mice, and test whether the microbiome effect is dosage and time dependent. Third, the study was a preclinical study. Given the microbiome are different between mice and humans, how AKBA changes the gut microbiome or microbial metabolites in human is of great interest.

*Boswellia serrata* is defined as “generally recognized as safe” by the United States Food and Drug Administration. As such, future research on the prebiotic effect of AKBA should be explored. There is already an established relationship between herbal medicine and an increase in *Akkermansia muciniphila*. Several Chinese medicines, including rhubarb, houttuynia, *Ganoderma lucidum*, and *Pueraria lobata*, have been shown to increase *Akkermansia muciniphila* [6]. With the prospects of more commercial food product use, AKBA’s antimicrobial properties should be further explored. The effect of bacterial load on the gut during long-term AKBA consumption, as well as the extent and persistence of AKBA changes to the gut microbiome, would be of interest.

## 5. Conclusions

To the best of our knowledge, this is the first work to demonstrate the effects of the *Boswellia serrata*-active compound AKBA on the gut microbiome in healthy mice. We observed different effects of AKBA consumption on the gut microbiome for both male and female mice, as well as the effects of the AKBA on blood metabolites. As AKBA has been observed to suppress *Akkermansia* growth and have no effect on *Bifidobacterium*, further work should be conducted on its direct effect on different bacteria and the gut microbiome as well as their mechanisms of action.

## Supplementary Materials

The following are available online at https://www.mdpi.com/article/10.3390/nu14040814/s1, Table S1: Differential abundant metabolites identified after 14 days treatment of AKBA.
