# Supplementary material for: Dietary Boswellia serrata Acid Alters the Gut Microbiome and Blood Metabolites in Experimental Models
Source: Nutrients. 2022 Feb 15;14(4):814. doi: 10.3390/nu14040814 (PMC8877038; doi:10.3390/nu14040814)
Supplement: Supplementary file 1 [file nutrients-14-00814-s001.zip › nutrients-1533677-supplementary.pdf]

Supplementary Table S1. **Differential abundant metabolites identified** after 14 days treatment of AKBA

Metabolite	m/z_ retention time (min)	I/D	P- Value	Possible formula	Possible compound	Possible formula	Possible compound
Male Feature Level							
1	163.0594__184.36	*Decrease*	1.08E-05	$C_4H_{10}N_3O_4$	2-(3-Amino-3-carboxypropyl)-1-hydroxy-1-oxohydrazinium; 1,1-Bis(2-hydroxyethyl)-3-oxo-2-triazanolate; N-[bis(2-hydroxyethyl)amino]-N-oxidonitrous amide	$C_{10}H_{12}S$	[1]Unidentifiable; 205 results found
2	174.0438__92.03	*Increase*	1.08E-05	$C_9H_7N_2O_2$	[1]Unidentifiable; 23 results found	$C_7H_7N_5O$	5-(3-amino-1H-1,2,4-triazol-5-yl)pyridin-3-ol
3	179.1289__271.38	*Increase*	0.00032	$C_8H_{20}O_4$	2-[2-(2-Methoxyethoxy)ethoxy]ethanol - methane (1:1); di(propylene glycol) methyl ether	$C_9H_{16}N_4$	N2-[2-(dimethylamino)ethyl]pyridine-2,5-diamine
4	148.0612__50.23	*Decrease*	0.00049	$C_5H_{11}NO_4$	[1]Unidentifiable; 351 results found	$C_3H_9N_4O_3$	1-Hydroxy-2-[N'-(2-hydroxyethyl)carbamimidoyl]-1-oxohydrazinium
Female Feature Level							
1	126.9198__34.12	*Decrease*	4.33E-05	Unidentifiable	Unidentifiable		
2	151.058__178.14	*Decrease*	8.66E-05	$C_3H_{10}N_3O_4$	Unidentifiable		
3	217.1336__196.45	*Increase*	0.00015	$C_{11}H_{16}N_5$	5-(2,2-Dimethylcyclopropyl)imidazo[4,5-b]pyridin-3-ium-1,3-diamine	$C_{10}H_{20}NO_4$	Aclatonium; (propionyloxy)-1-propanaminium
4	118.0548__32.78	*Decrease*	0.00015	$C_7H_6N_2$	Benzimidazole; Indazole; Phenyldiazomethane, (Diazomethyl) benzene		
5	155.0301__33.31	*Decrease*	0.00025	Unidentifiable	Unidentifiable		
6	163.0594__184.36	*Decrease*	0.00025	$C_4H_{10}N_3O_4$	2-(3-Amino-3-carboxypropyl)-1-hydroxy-1-oxohydrazinium; 1,1-Bis(2-hydroxyethyl)-3-oxo-2-triazanolate; N-[bis(2-hydroxyethyl)amino]-N-oxidonitrous amide	$C_{10}H_{12}S$	[1]Unidentifiable; 205 results found
7	107.0627__177.89	*Decrease*	0.00025	Unidentifiable	Unidentifiable		
8	146.0783__172.75	*Decrease*	0.00065	$C_7H_{15}OS$	Dimethyl(tetrahydro-2-furanylmethyl)sulfonium; 1-Isopropoxytetrahydrothiophenium		
9	202.1322__184.9	*Decrease*	0.00065	$C_9H_{19}N_2O_3$	[1]Unidentifiable; 14 results found		
10	164.0912__80.22	*Decrease*	0.00065	$C_6H_{15}NO_4$	[1]Unidentifiable; 38 results found		
11	190.0734__173.21	*Decrease*	0.00097	$C_{10}H_{11}N_2O_2$	[1]Unidentifiable; 62 results found	$C_3H_{11}N_8S$	Unidentifiable
12	200.0341__204.76	*Increase*	0.00145	$C_9H_5N_4O_2$	3-Cyano-7-methylpyrazolo[1,5-a]pyrimidine-6-carboxylate; 5-(5H-Tetrazol-5-ylidene)-3,5-dihydro-2-benzofuran-1-olate	$C_9H_{13}OS_2$	[1]Unidentifiable; 46 results found

[1] Unidentifiable with more than 4 results via ChemSpider databases are linked in supplementary data
